# Driving HIV-1 into a Vulnerable Corner by Taking Advantage of Viral Adaptation and Evolution

**DOI:** 10.3389/fmicb.2017.00390

**Published:** 2017-03-16

**Authors:** Shigeyoshi Harada, Kazuhisa Yoshimura

**Affiliations:** AIDS Research Center, National Institute of Infectious DiseasesTokyo, Japan

**Keywords:** HIV-1, antiretroviral therapy, neutralizing antibody, evolution, escape

## Abstract

Anti-retroviral therapy (ART) is crucial for controlling human immunodeficiency virus type-1 (HIV-1) infection. Recently, progress in identifying and characterizing highly potent broadly neutralizing antibodies has provided valuable templates for HIV-1 therapy and vaccine design. Nevertheless, HIV-1, like many RNA viruses, exhibits genetically diverse populations known as quasispecies. Evolution of quasispecies can occur rapidly in response to selective pressures, such as that exerted by ART and the immune system. Hence, rapid viral evolution leading to drug resistance and/or immune evasion is a significant barrier to the development of effective HIV-1 treatments and vaccines. Here, we describe our recent investigations into evolutionary pressure exerted by anti-retroviral drugs and monoclonal neutralizing antibodies (NAbs) on HIV-1 envelope sequences. We also discuss sensitivities of HIV-1 escape mutants to maraviroc, a CCR5 inhibitor, and HIV-1 sensitized to NAbs by small-molecule CD4-mimetic compounds. These studies help to develop an understanding of viral evolution and escape from both anti-retroviral drugs and the immune system, and also provide fundamental insights into the combined use of NAbs and entry inhibitors. These findings of the adaptation and evolution of HIV in response to drug and immune pressure will inform the development of more effective antiviral therapeutic strategies.

## Introduction

Human immunodeficiency virus type-1 (HIV-1) exhibits extremely high genetic diversity (Rambaut et al., 2004) indicating that rapidly changing genetic variation can confer on the virus the capacity to escape the immune system and anti-retroviral therapy (ART). The HIV-1 components presenting the highest degree of sequence diversity are the surface-expressed viral envelope glycoproteins (Env), which are prime targets for both entry inhibitors and neutralizing antibodies (NAbs) (Goulder and Watkins, 2008).

The function of Env is to facilitate the entry of HIV-1 into the target cell, a process mediated by recognition of the CD4 receptor and coreceptor (usually CCR5 or CXCR4) on the cellular membrane (Dalgleish et al., 1984; Klatzmann et al., 1984; Choe et al., 1996; Deng et al., 1996; Doranz et al., 1996; Dragic et al., 1996; Feng et al., 1996). Env is composed of the surface glycoprotein, gp120, and the transmembrane glycoprotein, gp41, which associate as a non-covalent complex to form a single subunit of a trimeric viral envelope spike (Wyatt and Sodroski, 1998). Gp120 is responsible for interactions with CD4 and the coreceptor, whereas gp41 anchors the Env machinery at the viral membrane and induces membrane fusion during viral entry (Freed and Martin, 1995; Bazan et al., 1998).

**FIGURE 1:**
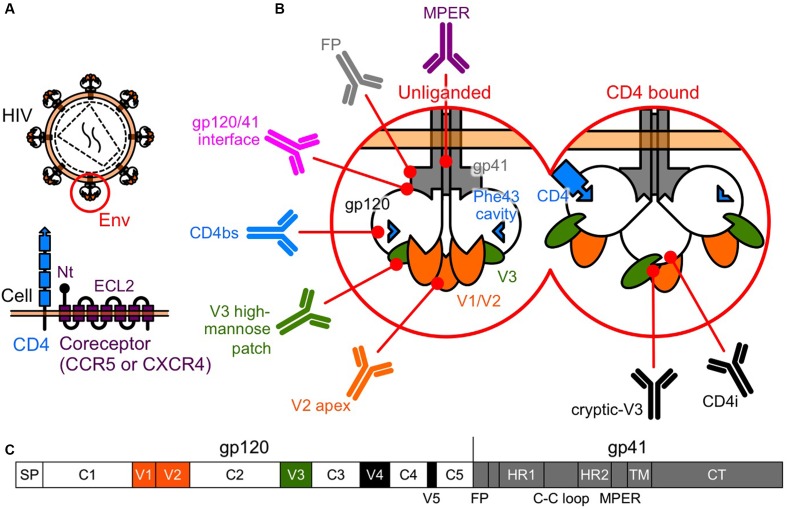
**Human immunodeficiency virus type-1 (HIV-1) Env. (A)** Entry of HIV-1 into a host cell involves interactions between the Env and the two-receptor mechanism of CD4 and the coreceptor. **(B)** Tertiary schematic view of HIV-1 Env. Following the binding of CD4 and gp120, gp120 undergoes conformational changes, moving from a rigid (unliganded) to a flexible state, allowing a subsequent interaction with the coreceptors. bNAbs have been identified that target the V2 apex, the V3 high-mannose patch, the CD4bs, the gp120/41 interface, the FP, and the MPER of gp41. In the CD4-bound state, a larger area is uncovered and potentially available for recognition by NAbs, such as V3-directed or CD4i, which recognize the conserved coreceptor-binding site. **(C)** Linear schematic view of HIV-1 Env. Gp120 is composed of five conserved regions (C1 to C5) that are interspersed with five variable regions (V1 to V5).

Many entry inhibitors have been developed to block the interaction of Env with the CD4 receptor, the coreceptor, or the fusion reaction. Currently, two entry inhibitors have been approved for clinical use, the fusion inhibitor, enfuvirtide (T-20) (Robertson, 2003), and the CCR5 inhibitor, maraviroc (MVC) (Dorr et al., 2005; Gulick et al., 2008). As with any anti-retroviral drug, HIV can develop resistance to T-20 and MVC. The major mechanism of resistance to T-20 is caused by mutations within the binding site on the HR1 region of gp41 (Greenberg and Cammack, 2004) (**Figure 1C**). On the other hand, clinical resistance to MVC involves different genetic alterations in *env* giving rise to highly divergent Env phenotypes (Roche et al., 2013). Potential molecular mechanisms of resistance to MVC include tropism switching to CXCR4-using (X4) viruses (Westby et al., 2006; Raymond et al., 2015), increased kinetics of the entry step (Reeves et al., 2002; Putcharoen et al., 2012), increased affinity for CD4 and/or CCR5 (Agrawal-Gamse et al., 2009; Pugach et al., 2009; Pfaff et al., 2010; Ratcliff et al., 2013), and utilization of MVC-bound CCR5 for entry (Pugach et al., 2007; Westby et al., 2007; Tilton et al., 2010; Roche et al., 2011).

In recent years, progress in identifying and characterizing highly potent broadly NAbs (bNAbs), has provided valuable templates for HIV-1 therapy and vaccine design (Kwong and Mascola, 2012; Kwong et al., 2013; Burton and Mascola, 2015; Burton and Hangartner, 2016). However, attempts to elicit such highly potent bNAbs by immunization have not been successful, due in part to the high genetic diversity of Env and the complex escape mechanisms employed by Env (Seaman et al., 2010).

Moreover, the replication capacity of HIV-1 is largely related to the efficiency of viral entry (Arts and Quinones-Mateu, 2003; Rangel et al., 2003). In this respect, evolutionary patterns of Env are important, and selective pressures exerted by NAbs and anti-retroviral drugs can contribute to its evolution. Thus, elucidation of these patterns would inform the development of more effective antiviral therapeutic strategies.

Recently, we investigated dynamic features of selective pressure on Env by assessing NAb sensitivities of HIV-1 escape mutants from MVC, and small-molecule CD4-mimetic compounds (CD4mc) that sensitize HIV-1 to NAbs. Thus, we summarize these recent advances and discuss the application of these findings to the development of more effective combinations of NAbs and anti-retroviral drugs.

## Fundamentals of HIV Entry

Entry of HIV-1 into a target cell involves interactions between Env and the two-receptor mechanism involving CD4 and the coreceptor. This interaction activates conformational changes in Env that lead to the membrane fusion reaction (Sattentau and Moore, 1995) (**Figure 1B**).

Gp120 is composed of five conserved regions (C1 to C5) that are interspersed with five variable regions (V1 to V5) (Starcich et al., 1986) (**Figure 1C**). The CD4 binding site (CD4bs) and especially the Phe 43 cavity, where Phe 43 of CD4 contacts gp120, are highly conserved among the different subtypes (Kwong et al., 1998). Following the binding of CD4 and gp120, the gp120 core undergoes conformational changes, moving from a rigid (unliganded) to a flexible state, allowing a subsequent interaction with the coreceptor (Myszka et al., 2000) (**Figure 1B**). Binding of gp120 to the coreceptor triggers further conformational changes in Env that fuse the viral membrane with the target cell membrane (Chan and Kim, 1998). Current models suggest the V3 tip interacts with the coreceptor second extracellular loop (ECL2), whereas the gp120 bridging sheet and the V3 stem interact with the coreceptor N terminus (Brelot et al., 1999; Farzan et al., 1999; Cormier and Dragic, 2002; Huang et al., 2005) (**Figure 1A**).

## Pressure of NAbs on the Evolution of Env

Recently, bNAbs have been isolated from HIV-1-infected individuals. Most major target specificities of these bNAbs have been mapped to various sites on Env, and include the V2 N160 glycan (V2 apex), the V3 N332 glycan (high-mannose patch), the CD4bs, the gp120/41 interface region, the fusion peptide (FP), and the membrane proximal external region (MPER) of gp41 (Burton and Mascola, 2015; Burton and Hangartner, 2016; Kong et al., 2016; van Gils et al., 2016). In addition, CD4 binding exposes highly conserved cryptic epitopes recognized by V3-directed or CD4-induced (CD4i) NAbs, which recognize the coreceptor binding site (Kwong and Mascola, 2012) (**Figure 1B**).

The V3-directed NAb, KD-247, is a humanized NAb with potent neutralizing activity. The epitope recognized by KD-247 was mapped to the IGPGR sequence of the V3-tip, which covers about half of subtype B. A phase-1b clinical study indicates that KD-247 reduces viral load in patients with chronic HIV-1 infection (Matsushita et al., 2015). However, HIV-1 can escape from the adaptive immune responses, and can become resistant to all anti-retroviral drugs. Therefore, in our previous *in vitro* study, we induced resistant variants against KD-247 using the JR-FL strain (Yoshimura et al., 2006). Resistance against KD-247 was associated with G314E substitution in the epitope on the V3-tip. Unexpectedly, the KD-247-resistant variant exhibited higher sensitivity to CCR5 inhibitors (TAK-779, aplaviroc and SCH-C) compared with the parental virus. Furthermore, our data showed strong synergistic interactions between KD-247 and CCR5 inhibitors (Yoshimura et al., 2006).

In addition to our studies, recent investigations of passive NAb therapy in HIV-infected individuals demonstrated that particular bNAbs could reduce levels of plasma viremia and suppress neutralization-sensitive viruses (Caskey et al., 2015; Lynch et al., 2015; Matsushita et al., 2015; Bar et al., 2016). However, a single use of NAbs could not suppress HIV completely and poses the danger of inducing escape variants *in vivo*. These findings suggest that combination strategies containing NAbs are needed to maintain virus suppression and prevent appearance of NAb-escape variants. Therefore, in the near future, combinations of NAbs and CCR5 inhibitors are likely to be efficient weapons against HIV-1.

## Effect of MVC-Resistance Mutations on Sensitivity to NAbs

The main mechanism of resistance to MVC appears to be related to changes in the V3 region, which enables the virus to utilize MVC-bound CCR5 coreceptors. The resistance is characterized by reductions in the maximal percent inhibition (MPI) value rather than shifts in the IC_50_ value (Pugach et al., 2007; Roche et al., 2011).

Pugach et al. noted that resistant variants against two CCR5 inhibitors (vicriviroc and AD101) were more sensitive to several types of NAbs compared with the parental virus (Pugach et al., 2008; Berro et al., 2009). Subsequently, we have reported the resistance induction of the primary KP-5P virus (subtype B, R5) against MVC *in vitro* (Yoshimura et al., 2014). Resistance to MVC was associated with V200I, T297I, K305R, and M434I substitutions near the CCR5 binding site. This MVC-resistant variant also exhibited extremely high sensitivity to three NAbs: b12 (CD4bs), 4E9C (CD4i), and KD-247. These results indicated that the MVC-resistance mutations might improve the accessibility of epitopes for the NAbs and, therefore, be incompatible with resistance to the NAbs (Yoshimura et al., 2014). More recently, Kuwata et al. (2015) showed that resistant variants against a CCR5 inhibitor, cenicriviroc, also became sensitive to three NAbs: VRC01 (CD4bs), 4E9C, and 0.5γ (V3).

**FIGURE 2:**
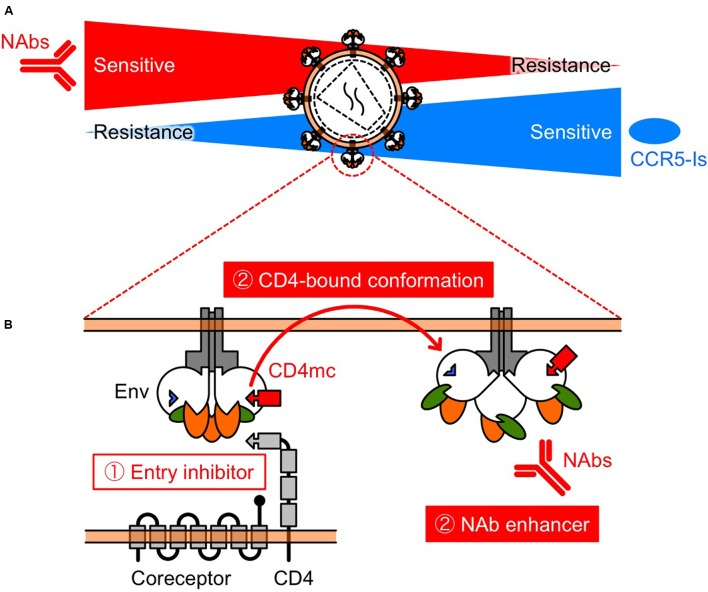
**Schematic view of the relationship between NAb resistance and CCR5 inhibitor resistance (A), and the function of CD4mc (B). (A)** Selection pressures on HIV-1 Env by NAbs and/or CCR5 inhibitors might turn the tide in the fight against HIV-1. The relationship indicates that NAbs and CCR5 inhibitors may restrict the emergence of variants that are resistant to each other. **(B)** CD4mc is a bifunctional entry inhibitor. The use of bifunctional entry inhibitors that display direct blockade of viral entry and exposure of epitopes to NAbs, should be effective in passive NAb immunization.

Another mechanism of resistance to MVC appears to be by a change in coreceptor tropism from CCR5 to CXCR4, or by the selection of minority variants of X4 or dual/mixed viruses (Westby et al., 2006). Indeed, Raymond et al. (2015) has reported that half of MVC-treated patients who experienced virological failure harbored X4 viruses at failure. Remarkably, previous studies have shown that early X4 variants are more sensitive to NAbs compared with their coexisting R5 variants (Ganesh et al., 2004; Lusso et al., 2005; Margolis and Shattock, 2006; Bunnik et al., 2007). In addition, increased CCR5 affinity is also a potential resistance mechanism, but we have shown that low-CCR5 affinity-adapted variants also became sensitive to CD4bs and CD4i NAbs (Yoshimura et al., 2014). Thus, several studies have demonstrated diverse resistance mechanisms against MVC, but all these resistance pathways might drive viral evolution into a corner, escape from which would require high sensitivity to NAbs. Moreover, these observations indicate that MVC and NAbs might limit the emergence of mutants that are resistant to each other, supporting the clinical use of combination therapy (**Figure 2A**).

However, it is not clear whether patients’ plasma IgG under MVC treatment can induce mutations in Env to enhance neutralizing activity. We are currently investigating the relationship between NAb responses and MVC treatment using patients’ plasma IgGs before and after MVC-containing combination ART (cART). Moreover, we think treatment with particular entry inhibitors and/or CD4mc can induce bNAbs *in vivo*; however, we await results for this prediction. Thus, we will perform experiments in animal models to induce or enhance NAbs using novel entry inhibitors and/or CD4mc treatment.

## Anti-Retroviral Pressure on the Selection of Env

Evolution of HIV-1 helps it to evade NAbs (Moore et al., 2012; Liao et al., 2013; Bouvin-Pley et al., 2014). cART, however, results in a reduction in the virus population size, which creates a genetic bottleneck. *In vivo* studies indicate that the bottleneck affects not only drug-target regions (e.g., reverse transcriptase), but also other regions of the viral genome, including the Env region (Zhang et al., 1994; Sheehy et al., 1996; Delwart et al., 1998; Nijhuis et al., 1998; Ibanez et al., 2000; Kitrinos et al., 2005; Charpentier et al., 2006; Nora et al., 2007). The population dynamics of the Env region might be important when bNAbs and novel entry inhibitors become available in the near future. However, it is hard to observe effects of an anti-retroviral drug-induced bottleneck on the Env region *in vivo*.

Thus, we induced variants against anti-retroviral drugs using primary swarm isolates (Harada et al., 2013). As a result, the phylogenetic clustering of raltegravir (an integrase inhibitor)-, lamivudine (a reverse transcriptase inhibitor)- and saquinavir (a protease inhibitor)-induced variants was entirely distinct from that of non-drug-treated controls. Among these drug-induced variants, the variable regions of gp120 were very similar to each other. Conversely, the non-drug-treated variant was quite different from the drug-induced variants. These results imply that, under selective pressure of non-entry inhibitors, the virus may choose a representative Env sequence from the viral population to gain a growth advantage (Harada et al., 2013). In addition to our results, a supporting study by Mesplede et al. (2015) showed that treatment with dolutegravir (an integrase inhibitor) results in a reduction in viral genetic diversity. Further studies are needed to confirm our observations, but these results may provide a new paradigm for viral evolution in the novel NAb plus anti-retroviral drug combination therapy era.

### CD4mc CAN EXPOSE HIV-1 NEUTRALIZATION EPITOPES

Binding of CD4 to gp120, is the first essential step of the entry process. The multiple contacts made by Phe 43 and Arg 59 of CD4 with gp120 residues in CD4bs contribute significantly to CD4–gp120 binding (Kwong et al., 1998). The critical Phe 43 of CD4 becomes buried in a binding pocket of gp120, termed the Phe 43 cavity. This cavity is known to be highly conserved among the different subtypes and is therefore considered a particularly interesting target for inhibitors of CD4–gp120 interaction (Kwong et al., 1998).

Molecules that mimic the CD4 receptor, such as soluble CD4 (sCD4), CD4 immunoadhesin (CD4-Ig), sCD4 mini-proteins, and CD4mc have been developed (Vita et al., 1999; Grupping et al., 2012). sCD4, CD4-Ig, and sCD4 mini-protein have been studied as potential therapeutics (Smith et al., 1987; Fisher et al., 1988; Hussey et al., 1988; Jacobson et al., 2000; Fletcher et al., 2007; Dereuddre-Bosquet et al., 2012; Gardner et al., 2015). These studies in patients and non-human primate models have provided proof of principle that viral entry can be successfully blocked *in vivo*. In particular, Gardner et al. (2015) demonstrated that eCD4-Ig, a fusion of CD4-Ig with a small CCR5-mimetic peptide, was on average more potent, and much broader than bNAbs. Moreover, adeno-associated virus-delivered eCD4-Ig, provided durable protection for immunized monkeys against high-dose intravenous SHIV challenge (Gardner et al., 2015).

The prototype of CD4mc, NBD-556, was identified in a screen for inhibitors of the CD4–gp120 interaction (Zhao et al., 2005). We and others have been exploring the potential of NBD-556-derived CD4mc as a novel class of HIV entry inhibitor (Madani et al., 2004, 2008, 2014, 2016, 2017; Narumi et al., 2010, 2011, 2013; Yamada et al., 2010; Yoshimura et al., 2010; Lalonde et al., 2011, 2012, 2013; Courter et al., 2014; Richard et al., 2015; Melillo et al., 2016; Mizuguchi et al., 2016; Ohashi et al., 2016). The binding of CD4mc in the Phe 43 cavity blocks CD4-gp120 interaction and, induces conformational changes in gp120 similar to those observed upon sCD4 binding (**Figure 2B**) (Schon et al., 2006; Haim et al., 2009; Curreli et al., 2014; Kwon et al., 2014). sCD4 significantly enhance neutralization by CD4i (Thali et al., 1993) and some V3 NAbs (Lusso et al., 2005). Remarkably, CD4i and V3 NAbs are present in HIV-infected individuals during the early stage of infection (Decker et al., 2005). Consequently, we hypothesized that CD4mc can cause exposure of cryptic epitopes to antibodies, allowing virus neutralization. As a result, combinations of CD4mc (NBD-556 or YYA-021) with CD4i or V3 NAbs produced strong synergistic antiviral interactions (Yamada et al., 2010; Yoshimura et al., 2010) (**Figures 1**, **2B**). Moreover, we found that CD4mc sensitized a clinical isolate to autologous plasma antibodies from the same time point (Yoshimura et al., 2010).

Recently, this approach has been extended to combining vaccine with CD4mc. In studies using prototypic CD4mc BNM compounds, Madani et al. (2014, 2016) demonstrated that CD4mc sensitized the virus to antibodies elicited by immunization of humans and monkeys. These studies establish the proof of concept that CD4mc can sensitize primary viruses to antibodies that are present in plasma of infected or vaccinated individuals. In addition, Richard et al. (2015) reported that CD4mc could efficiently sensitize primary CD4 T cells from HIV-1-infected individuals to antibody-dependent cell-mediated cytotoxicity (ADCC) mediated by autologous sera and effector cells.

Based on these results, further studies are needed to investigate the effectiveness of delivery methods of CD4mc. Small molecules such as CD4mc have many advantages over conventional immunotherapeutic agents, including ease of production and the potential for oral administration. Furthermore, the use of bifunctional entry inhibitors that display direct blockade of viral entry and exposure of epitopes to NAbs should be effective in passive NAb immunization.

## Conclusion

Extensive genetic diversity in the Env region presents significant obstructions to the development of promising therapies and vaccines against HIV-1. However, selection pressures on the Env region by NAbs, entry inhibitors, and/or non-entry antiviral inhibitors, might turn the tide in the fight against HIV-1. Moreover, bifunctional entry inhibitors such as CD4mc might potentiate these selection pressures. Thus, by taking advantage of the adaptation and evolution of HIV resulting from drug and immune pressure, we might drive HIV-1 into a vulnerable corner.

## Author Contributions

All authors listed, have made substantial, direct and intellectual contribution to the work, and approved it for publication.

## Conflict of Interest Statement

The authors declare that the research was conducted in the absence of any commercial or financial relationships that could be construed as a potential conflict of interest.
